# Matrine induces caspase-independent program cell death in hepatocellular carcinoma through bid-mediated nuclear translocation of apoptosis inducing factor

**DOI:** 10.1186/1476-4598-13-59

**Published:** 2014-03-16

**Authors:** Huan Zhou, Minying Xu, Ya Gao, Zhigang Deng, Hanwei Cao, Wenqing Zhang, Qiao Wang, Bing Zhang, Gang Song, Yanyan Zhan, Tianhui Hu

**Affiliations:** 1 Cancer Research Center, Xiamen University Medical college, Xiamen 361102, China; 2 Department of General Surgery, Mianyang Central Hospital, Mianyang 621000, China; 3 Department of Basic Medicine, Xiamen University Medical college, Xiamen 361102, China

**Keywords:** Hepatocellular carcinoma, Matrine, Caspase-independent PCD, Bid, AIF

## Abstract

Matrine, a clinical drug in China, has been used to treat viral hepatitis, cardiac arrhythmia and skin inflammations. Matrine also exhibits chemotherapeutic potential through its ability to trigger cancer cell death. However, the mechanisms involved are still largely unknown. The objective of this study was to investigate the major determinant for the cell death induced by matrine in human hepatocellular carcinoma. We use human hepatocellular carcinoma cell line HepG2 and human hepatocellular carcinoma xenograft in nude mice as models to study the action of matrine in hepatocellular cancers. We found that caspase-dependent and -independent Program Cell Death (PCD) occurred in matrine-treated HepG2 cells, accompanied by the decreasing of mitochondrial transmembrane potential and the increasing ROS production. Further studies showed that AIF released from the mitochondria to the nucleus, and silencing of AIF reduced the caspase-independent PCD induced by matrine. What’s more, AIF nuclear translocation, and the subsequent cell death as well, was prevented by Bid inhibitor BI-6C9, Bid-targeted siRNA and ROS scavenger Tiron. In the *in vivo* study, matrine significantly attenuated tumor growth with AIF release from mitochondria into nucleus in nude mice. These data imply that matrine potently induce caspase-independent PCD in HepG2 cells through Bid-mediated AIF translocation.

## Background

Hepatocellular carcinoma (HCC) is the fifth most common malignancy [1], and the third cause of cancer-related mortality worldwide [2]. The overall incidence of HCC is fairly high and steadily rising in both the developed and the developing world, on account of the prevalence of the two major risk factors, hepatitis B virus (HBV) and hepatitis C virus (HCV) [3]. Despite the poor understanding of how HBV and/or HCV lead to HCC, some antiviral agents have been found to exhibit anticarcinogenic effects [4]. Matrine, an alkaloid isolated from the root of Sophora subprostrata, is originally used in the treatment of enteritis, hepatitis, hepatic fibrosis and hypertension in China [5]. Matrine is also found to induce cell death in many kinds of cancer cells, including cervical cancer, leukemia, gastric cancer, lung cancer and breast cancer [6-10], and thus is considered as a promising drug for cancer therapy.

Caspase-dependent apoptosis is the best-known modality of programmed cell death (PCD) [11]. Conventional anticancer agents, regardless of their distinct targets and mechanisms, primarily induce cell death via caspase-dependent apoptosis [12,13]. Meanwhile, cancer cells are usually sensitive to caspase-dependent apoptotic induction initially, but eventually they become drug-resistant due to the dysregulation of apoptotic machinery, manifested as the over-expression of anti-apoptotic proteins and the defects in pro-apoptotic factors [14,15]. Therefore, developing new drugs and methods that can specifically treat drug-resistant cancers is an urgent task for saving lives.

Fortunately, increasing evidence shows that PCD can also happen in the presence of the pan-caspase inhibitor z-VAD-fmk [16,17]. In C. elegans, some cells succumb to developmental cell death in a caspase-independent pathway [18]. N-methyl-N’-nitro-N-nitrosoguanidine (MNNG) can also induce caspase-independent PCD (ciPCD) in MEF cells [19]. However, understanding of how cells actually die during ciPCD remains limited. Recently, strong evidence of the important role of AIF in ciPCD, via its releasing from mitochondria into nucleus [20,21], has been shown in studies performed with alkylating DNA damage agents [22,23]. Despite the prominent role of AIF in ciPCD signaling, the mechanisms upstream of AIF releasing are still poorly defined. Previous studies have suggested that a member of the bcl-2 family, Bid, facilitates the insertion of Bak or Bax into mitochondrial membrane to form functional oligomers, which results in the depolarization of the inner mitochondrial membrane and the subsequent AIF translocate from mitochondria to nucleus [24,25]. Thus, Bid regulates AIF translocation is involved in ciPCD, and might be a potential mechanism for developing effective drug to induce cancer cell death. HepG2 cell line is well characterized of hepatocellular carcinoma and is widely used in the study [26,27]. Therefore, we investigated the mechanisms of matrine-induced cell death in hepacellular carcinoma cell line HepG2 *in vitro* and subcutaneous xenograft tumors in nude mice *in vivo*. We found that caspase-dependent and caspase-independent PCD occurred in HepG2, accompanied by mitochondrial transmembrane potential losing, ROS production, cytochrome c and AIF released from the mitochondria. What’ more, AIF was required in caspase-independent cell death and its translocation was prevented by Bid inhibitor BI-6C9, Bid siRNA and ROS scavenger Tiron. In the *in vivo* study, matrine significantly attenuated tumor growth with AIF release from mitochondria and accumulation in nucleus in nude mice. These findings suggest that matrine may provide a new selectivity for hepatocarcinoma therapy through the induction of AIF-mediated ciPCD.

## Results

### Matrine induced caspase-dependent and -independent PCD in HepG2 cells

**Figure 1:**
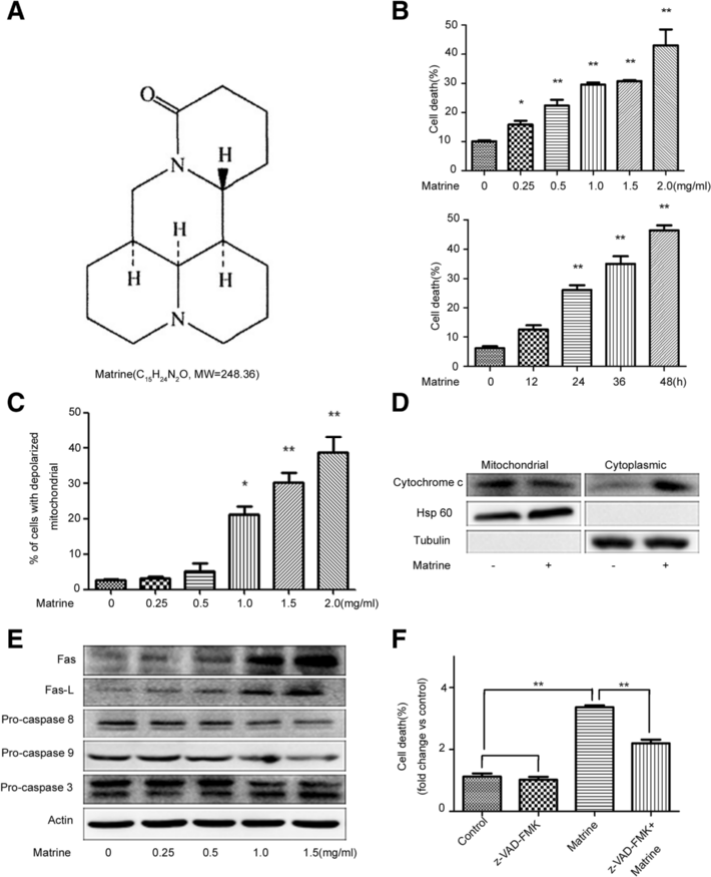
**Matrine induced caspase**-**dependent and** -**independent cell death in HepG2 cells. (A)** The chemical structure of matrine. **(B)** Cells were treated with different concentrations of matrine (0, 0.25, 0.5, 1, 1.5, 2 mg/ml) for 24 hrs, or 1.5 mg/ml matrine for different time periods (0, 12, 24, 36, 48 hrs), and then the cell death was determined by Annexin V/PI staining assay. **(C)** Cells were collected after treated with different concentrations of matrine for 24 hrs and analyzed for ΔΨm by Rh123 retention. **(D)** Expression of cytosolic and mitochondrial cytochrome c in HepG2 cells with matrine treatment. Cells were treated with 1.5 mg/ml of matrine for 24 hrs. The cytoplasmic and mitochondrial proteins were segregated and analyzed by western blot assay. **(E)** HepG2 cells were treated with different concentrations of matrine for 24 hrs. The total protein was extracted and the expression of apoptosis-related proteins was analyzed by western blot assay. **(F)** Effect of pancaspase inhibitor, z-VAD-fmk, on matrine-induced cell death. HepG2 cells were pretreated with z-VAD-fmk (20 μM, 2 hrs) before matrine treatment. Cell death was analyzed by Annexin V/PI staining assay. Data are presented as means ± S.D. of three separate experiments. The basal level of cell death was normalized to 1. *p < 0.05, **p < 0.01 *vs*. control.

To investigate the mode of cell death induced by matrine (structure in Figure 1A) treatment in HepG2 cells, flow cytometry was employed. Results showed that the cell death rate was increased in both dose- and time-dependent manners (Figure 1B & Additional file 1: Figure S1A), with the decreasing of mitochondrial transmembrane potential (ΔΨm) (Figure 1C & Additional file 1: Figure S1B) and the releasing of cytochrome c from mitochondria (Figure 1D). These results are consistent with previous report in AML cells [9] and our other results in QBC939 (Xu and Hu, unpublished data). Additionally, matrine treatment increased the levels of apoptosis-related proteins Fas and Fas-L and the cleaved caspase-3, while decreased the levels of procaspase-8, procaspase-9 and procaspase-3 (Figure 1E). Notably, the pathways of cell death induced by matrine include caspase activation.

Cell death can also happen through an alternative mitochondrial pathway, which is independent of caspase activation [20]. To elucidate whether the caspase-independent pathway was also involved in the matrine-induced cell death, a pancaspase inhibitor z-VAD-fmk (Calbiochem), which completely inhibited the caspase-dependence of TNF-induced cell death in A cells [28] and TNF/cycloheximide-induced cell death in colon tumour cells [23], was used. The results showed that cell death induced by matrine was partly suppressed by z-VAD-fmk, compared with the control group (Figure 1F & Additional file 1: Figure S1C). Thus, we can conclude that matrine-induced cell death through caspase-dependent and -independent pathway in HepG2 cells.

### AIF translocation is required for matrine-induced ciPCD in HepG2 cells

**Figure 2:**
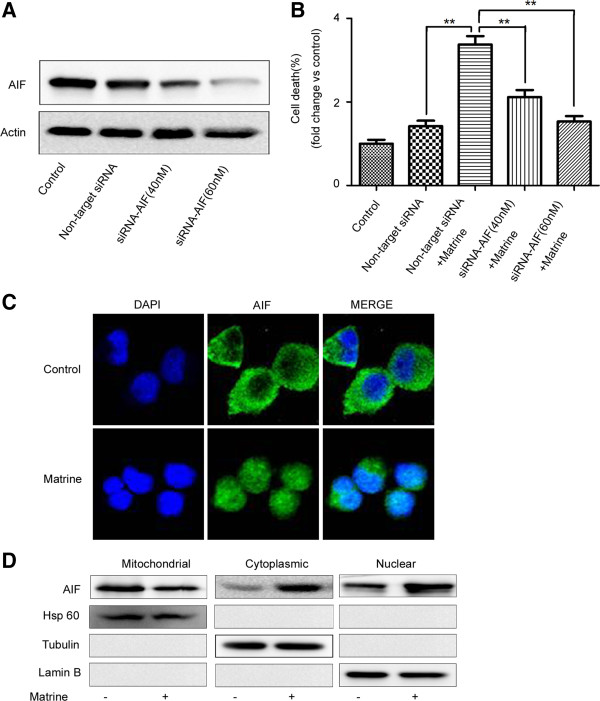
**AIF translocation is required for matrine**-**induced ciPCD. (A-B)** HepG2 cells were transfected with AIF siRNA (40 or 60 nM) or non-targeted siRNA for 24 hrs, and then treated with matrine at 1.5 mg/ml for 24 hrs. **(A)** AIF expression levels were detected by western blot analysis using an anti-AIF antibody. Actin was used as a protein loading control. **(B)** Cells were stained with Annexin V/PI to assess cell viability by flow cytometry. The basal level of cell death was normalized to 1.*p < 0.05, **p < 0.01 *vs*. non-target siRNA + Matrine. **(C)** Immunostaining was performed to localize AIF with an anti-AIF antibody and a FITC-conjugated secondary antibody (green). The nuclei were detected using DAPI staining (blue). **(D)** Cytoplasmic, nuclear, and mitochondrial fractions were prepared for western blot analysis using anti-AIF, anti-β-tubulin (cytosolic marker), anti-Hsp60 (mitochondrial marker) and anti-Lamin B (nuclear marker) antibodies.

AIF was reported to play important roles in ciPCD [20]. To further study how matrine induce ciPCD in HepG2 cells, we knocked down endogenous AIF in HepG2 cells by transfecting AIF-targeted siRNA (Figure 2A). We found that down-regulation of AIF expression effectively attenuated matrine-induced ciPCD in HepG2 cells, compared to the cells transfected with a non-targeted siRNA (Figure 2B & Additional file 2: Figure S2), indicating the important role of AIF in matrine-induced ciPCD.

The translocation of AIF from the mitochondria to the nucleus is required for its activation in ciPCD [29], so we combined immunostaining and cell fractionation methods to detect whether matrine affected the subcellular location of AIF. DAPI was used to stain nucleus. AIF showed a granular pattern in the mitochondria, whereas after treatment with matrine, AIF was detected in the nucleus (Figure 2C). Moreover, we confirmed AIF location by western blot analysis of fractioned cellular components (Figure 2D). These results indicated that AIF release to the cytoplasm and translocate to the nucleus by matrine treatment in HepG2 cells.

### Bid plays an essential role in matrine-induced AIF translocation

**Figure 3:**
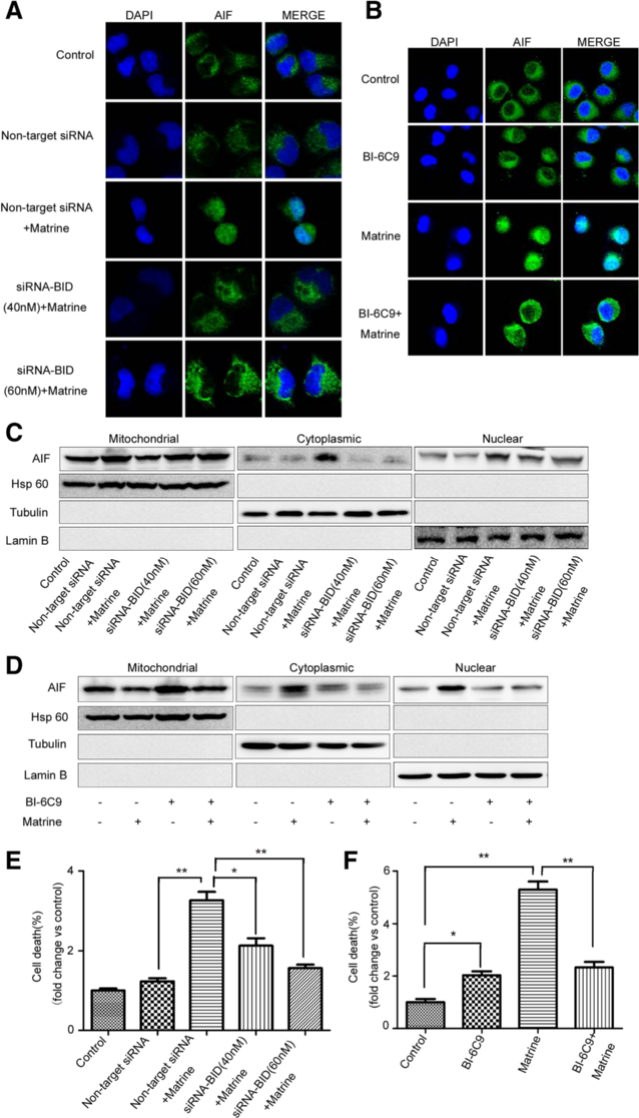
**Bid mediated the cell death induced by matrine**, **as an upstream regulator of AIF.** HepG2 cells were transfected with Bid siRNA (40 or 60 nM) or non-targeted siRNA for 24 hrs **(A**, **C**, **E)** or pretreated Bid inhibitor BI-6C9 (10 μM) for 1 hr **(B, D, F)**, then treated with matrine at 1.5 mg/ml for 24 hrs. **(A**, **B)** Immunostaining was performed to localize AIF with an anti-AIF antibody and a FITC-conjugated secondary antibody (green). The nuclei were detected using DAPI staining (blue). **(C**, **D)** Cytoplasmic, nuclear, and mitochondrial fractions were prepared for western blot analysis to localize AIF with an anti-AIF antibody using anti-AIF, anti-β-tubulin (cytosolic marker), anti-Hsp60 (mitochondrial marker) and anti-Lamin B (nuclear marker) antibodies. **(E**, **F)** Cells were stained with Annexin V/PI to assess cell viability by flow cytometry. The basal level of cell death was normalized to 1.*p < 0.05, **p < 0.01 *vs*. non-target siRNA + Matrine **(E)**, Matrine **(F)**.

How is AIF activated by matrine in HepG2 cells? A manuscript has already described that Bid regulates AIF-mediated caspase-independent necroptosis [25]. Thus, we transfected HepG2 cells with Bid siRNA or pretreated with Bid inhibitor (Santa Cruz Biotechnology), and then, analyzed AIF location by immunostaining and western blotting. Down-regulation of Bid expression significantly reduced matrine-induced AIF nuclear translocation in HepG2 cells (Figure 3A). Moreover, BI-6C9 also blocked matrine-induced AIF translocation detected by confocal microscopy (Figure 3B). The findings were confirmed with the results from western blotting (Figure 3C &3D). We can conclude that Bid was required for matrine-induced AIF release from the mitochondria to the nucleus.

To examine whether Bid inhibition attenuates the cell death induced by matrine in HepG2, we performed flow cytometry analysis. Down-regulation of Bid expression attenuated matrine-induced cell death by transfecting Bid siRNA (Figure 3E & Additional file 3: Figure S3A). In addition, Bid inhibitor BI-6C9 was applied to confirm the essential role of Bid in cell death induced by matrine. Pretreatment with BI-6C9 rescued matrine-induced cell death in HepG2 (Figure 3F & Additional file 3: Figure S3B). Thus, activation of Bid may serve a mechanism by which matrine induces PCD through regulation of AIF nulear translocation in HepG2 cells.

### The role of AIF in matrine-induced cell death partly occurs through increasing energy metabolism-associated ROS production in HepG2

**Figure 4:**
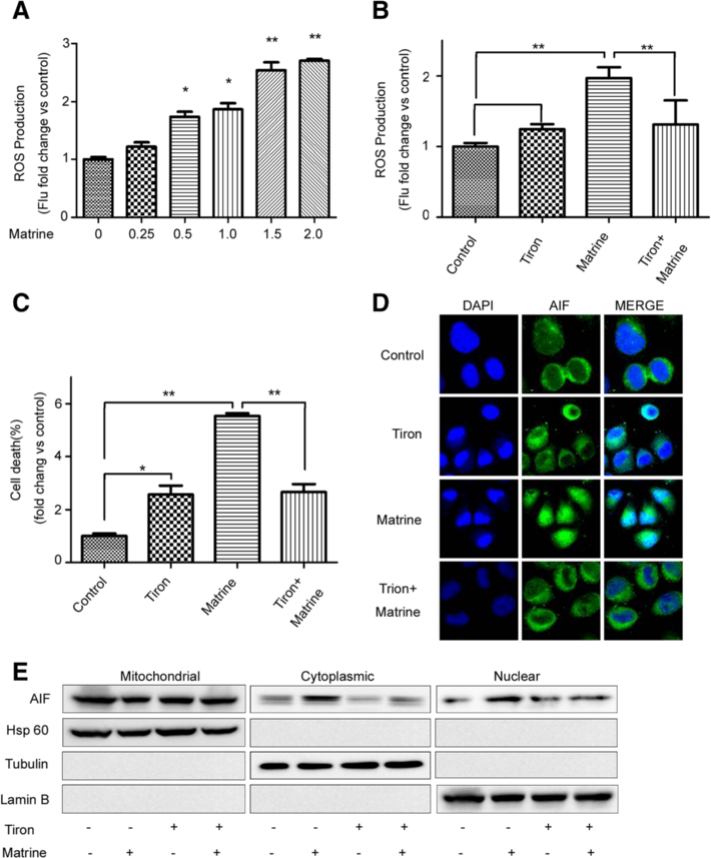
**ROS production is required for AIF activation in HepG2 cells. (A**, **B)** Cells were treated with matrine at different concentrations (0, 0.25, 0.5, 1, 1.5, 2 mg/ml) for 24 hrs or at 1.5 mg/ml for 24 hrs in the absence or presence of Tiron (5 μM), the ROS scavenger. ROS levels were then detected by flow cytometry. **(C)** Tiron significantly attenuated matrine-induced cell death as determined by Annexin V/PI staining and flow cytometry. The basal level of cell death was normalized to 1.*p < 0.05, **p < 0.01 *vs*. Matrine. Immunostaining **(D)** and western blot analyses **(E)** demonstrated that matrine-induced AIF translocation to the nucleus was inhibited by Tiron.

The apoptosis/necrosis should at least partly occur through increasing energy metabolism-associated ROS production [28]. ROS served as a possible upstream or downstream mediators of cell death [30]. Thus, we detected ROS production using dihydroethidium (Molecular Probe), a cell-permeable fluorescence dye that reacts with a broad spectrum of ROS. Flow cytometry assay shows that matrine treatment increase ROS production in a dose-dependent manner (Figure 4A & Additional file 4: Figure S4A). We further analyzed whether the inhibition of ROS influenced matrine-mediated cell death. Pretreatment with a cell permeable ROS scavenger, Tiron (Santa Cruz Biotechnology), blocked ROS generation and attenuated cell death induced by matrine (Figure 4B, C & Additional file 4: Figure S4B, C ), suggesting that ROS generation could mediate PCD induced by matrine in HepG2 cells.

To investigate the role of ROS in AIF activation, pretreatment of HepG2 cells for 24 hrs with Tiron. We combined immunofluorescence and cell fractionation methods to detect the AIF location. Immunofluorescence results show that Tiron inhibited matrine-induced AIF translocate from mitochondria into nucleus (Figure 4D). Moreover, we confirmed this finding by western blotting of fractioned cellular components (Figure 4E).

### Matrine inhibits the proliferation of HepG2 cells in the nude mice

**Figure 5:**
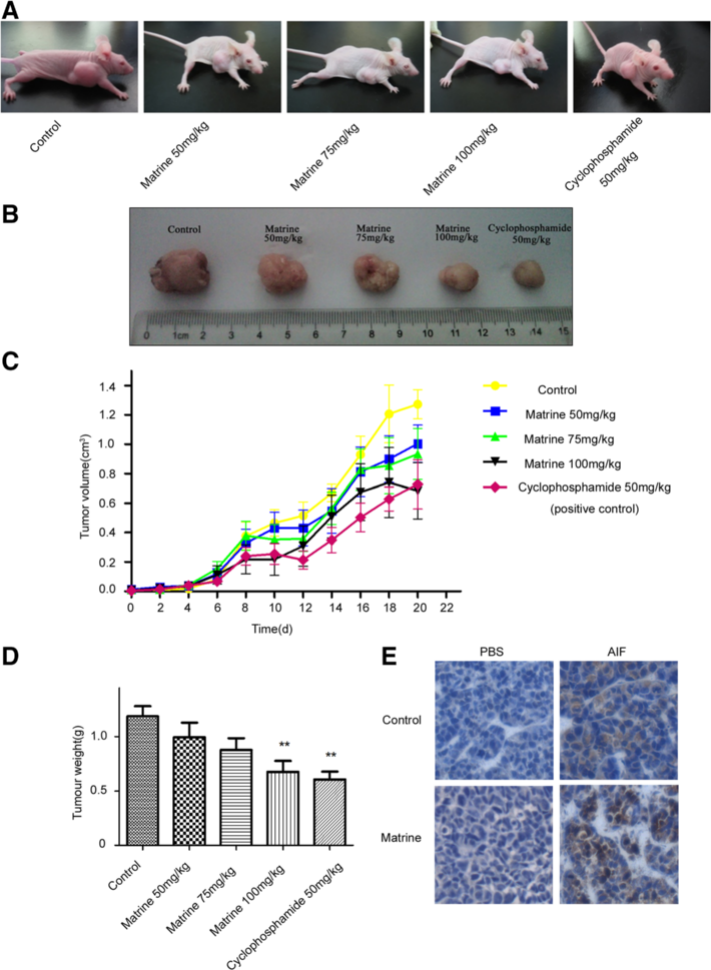
**Matrine inhibits the proliferation of HepG2 cells in nude mice. (A**, **B)** Typical photographs of tumor samples from the nude mice treated with different doses of matrine. HepG2 cells were implanted by subcutaneous injection into the right fore leg of the nude mice. Different doses of matrine were administered to the mice by subcutaneous injection every other day. Mice in control group were given physiological saline alone. After 3 weeks, the mice were sacrificed. **(C**, **D)** Volume and Weight of tumor samples from nude mice treated with different doses of matrine. Data are reported as means ± S.D. of three separate experiments. *p < 0.05, **p < 0.01 *vs*. control. **(E)** Subcellular location of AIF in tumor samples from the control group and the 100 mg/kg matrine-treated group were analyzed by immunohistochemistry.

We established the model of hepatocellular carcinoma cells xenograft in nude mice by injecting HepG2 cells. When the tumors reached around 0.120 cm^3^, the mice were randomly assigned to five groups to receive intraperitoneally injection of saline (negative control), 50, 75, 100 mg/kg of matrine, or 50 mg/kg of cyclophosphamide (positive control) every two days. As show in Figure 5A-D, matrine inhibited the proliferation of HepG2 cells in nude mice.

The subcellular location of AIF in the subcutaneous tumor tissues were further examined by immunohistochemical staining. AIF located in the cytoplasm in the tumors from negative control group, while tumors from 100 mg/kg matrine treatment group showed a predominantly nuclear localization of AIF (Figure 5E), in accordance with the results of HepG2 cells treated with matrine *in vitro*.

## Discussion

Many traditional Chinese medicine and related active compounds have been reported to have potent anti-cancer activities, such as matrine. Current studies have proposed that the anti-carcinogenic property of matrine is closely associated with inhibition of proliferation and induction of apoptosis [6-8]. Matrine induces apoptosis in U937 cells and K562 cells via the mitochondrial pathway, a cytochrome c-triggered caspase activation pathway [9,31]. In accordance with previous observations in a variety of tumour cell lines, we found that matrine induced cell death partly through the caspase-dependent pathway in HepG2 cells. The matrine-induced cell death in HepG2 cells appeared to accompany with the release of cytochrome c, ROS and AIF, together with the decreasing of mitochondrial transmembrane potential, which indicate a critical role for mitochondria in matrine-induced cell death. It is worth notice that cytochrome c is involved in caspase-dependent pathway, ROS and AIF are mostly associated with caspase-independent pathway [20], so we tested if caspase independent pathway is also involved. As expected, we found that z-VAD-fmk, an inhibitor of caspase activation, did not block all of cell death upon matrine treatment in HepG2 cells, suggesting the existence of ciPCD. ROS generation may be a critical inductor in matrine-induced ciPCD because the free radical scavenger Tiron blocked both cytotoxicity and AIF mitochondria-nucleus translocation. Thus, matrine induced concomitantly caspase-dependent and caspase-independent death in HepG2 cells.

The discovery and understanding of ciPCD will open new perspectives for the treatment of tumor. On the one hand, the existence of caspase-independent pathways provides new options to kill tumor cells [23,32], and one of such therapies has already advanced into clinical trials [33,34]. On the other hand, because the anti-apoptotic mechanisms that suppress caspases activation have also been shown to be involved in drug resistance of tumor cells [15,35], combination of caspase-directed and alternative therapies based on caspase-independent death effectors will provides a more efficient approach to circumvent the commonly observed therapy resistance of transformed cells.

It was reported that truncated Bid (tBid), the cleaved and active form of Bid, translocates to the mitochondria and activates Bax/Bak homooligomerization in the outer mitochondrial membrane, followed by the release of AIF from mitochondria in MNNG-induced ciPCD [24]. We did found that matrine decreased the levels of Bid in the cytosol and the nucleus (data not shown), inferring the cleavage of Bid into tBid. However, the mitochondrial Bid did not obvious increased under our experimental conditions (data not shown), which might be due to the short half-life of tBid. And we further found that transfected HepG2 cells with Bid siRNA or pretreated with Bid inhibitor prevented matrine-induced AIF nuclear translocation. Recently, necroptosis, a parallel but nonapoptotic pathway of cell death, has been described [36-38]. After MNNG treatment, PARP-1 activation stimulates the release of AIF from the mitochondria [39,40], which depends on calpain Cys proteases and the proapoptotic Bcl-2 member Bid and Bax [24,25]. And then AIF rapidly translocalizes to the nucleus, in cooperation with histone H2AX [19,22] and cyclophilin A [41], provokes caspase-independent necroptosis [20]. Moreover, ROS is essential for this type of cell death [28]. Matrine induced ciPCD through Bid-mediated AIF nuclear translocation and ROS production were in accord with the signaling pathway of MNNG-induced caspase-independent necroptosis. Therefore, the signaling pathway of MNNG-induced caspase-independent necroptosis might be the mode of cell death HepG2 cells following treatment with matrine.

## Conclusion

The present studies demonstrated that matrine can induce concomitantly caspase-dependent and caspase-independent cell death in HepG2 cells through Bid-regulated AIF (the best studied example of a ciPCD mediator) nuclear translocation pathway. The elucidation of the mechanism of matrine-induced ciPCD suggests a possible therapeutic role of matrine and its potential of combination with other chemotherapies targeting caspase-dependent pathway to treat heptacellular carcinoma.

## Methods

### Cell culture

The human hepatocellular carcinoma cell line HepG2 (obtained from Shanghai Institute of Cell Biology, Chinese Academy of Sciences, Shanghai, China) were cultured in DMEM supplemented with 10% fetal bovine serum, penicillin-streptomycin, in a humidified 5% CO2 atmosphere. The HepG2 cells in mid-log phase were used in experiments. Matrine was purchased from Sigma Chemical Co. (St. Louis, MO, USA), dissolved in distilled water at a concentration of 50 mg/ml as a stock solution and stored at 4°C in the dark.

### Transient transfection of siRNA

Bid siRNA, AIF siRNA and nonfunctional control siRNA were purchased at Santa Cruz Biotechnology. HepG2 cells were transfected with non-targeted, Bid siRNA or AIF siRNA using Lipofectamine 2000 (Life Technology, Carlsbad, CA, USA), according to the manufacturer’s instructions. After 24 hrs transfection, cells were treated with matrine in serum-starved medium for 24 hrs for cell death assay and western blot analysis to determine the expression level of AIF.

### Flow cytometery

We used Annexin V-FITC (0.1 mg/ml) for the assessment of phosphatidylserine exposure, propidium iodide (PI, 0.5 mg/ml) for cell viability analysis, Rh123 (50 nM) for DCm quantification and DCFH-DA (10 μM) for detecting ROS production. The samples were analyzed by flow cytometry (Becton Dickinson FACS can).

### Immunostaining

After matrine treatment, cells were washed with PBS and fixed with 4% paraformaldehyde for 15 min at room temperature. Cells were permeabilized using 2% Triton X-100 for 5 min in PBS at room temperature and blocked in 3% bovine serum albumin in PBS containing 1% Triton X-100 for 1 hr at room temperature. Slides were then incubated with goat monoclonal antibody against AIF (1:50) over night at 4°C. The cells were then washed and subsequently incubated with both FITC-conjugated rabbit anti-goat secondary antibodies at a dilution of 1:50 for 1 hr at room temperature. Cells were mounted in antifade solution onto glass slides and observed under confocal microscopy.

### Cytosolic, nuclear and mitochondrial protein isolation

After matrine treatment, cells were collected and resuspended in five volumes of ice-cold extract Buffer A (20 mmol/L HEPES-KOH, pH7.5, 1.5 mmol/L MgCl_2_,1 mmol/L EDTA,1 mmol/L EGTA,1 mmol/L DTT, 0.1 mmol/L PMSF) and were homogenized. The homogenates were centrifuged at 1000 × *g* for 10 min, 4°C. The supernatant was collected and centrifuged at 10,000 × *g* for 15 min, to obtain the mitochondria pellets (resuspended in 100 μL Buffer A). The supernatants were further centrifuged at 100,000 × *g* for 1 hr (4°C) to collect the cytosolic fraction. For the isolation of the nuclear proteins, ice-cold extract NE-Buffer A (10 mmol/L HEPES, pH7.9, 1.5 mmol/L MgCl_2_,10 mmol/L KCl, 0.5 mmol/L DTT, 0.5 mmol/L PMSF) was added to the pellet after 1000 × *g* centrifugation. The pellet and NE-Buffer A were mixed by gently pipetting and kept on ice for 15 min. The mixture was then treated with 10% NP-40, vortexed for 10 sec, and centrifuged at 10,000 × *g* for 30 sec, 4°C. The supernatant was discarded and the pellet was resuspended and homogenized in NE-Buffer B (5 mmol/L HEPES, pH7.9, 26% Glycerol, 1.5 mmol/L MgCl_2_, 0.2 mmol/L EDTA, 0.5 mmol/L DTT, 0.5 mmol/L PMSF), and then centrifuged at 13,000 × *g* for 20 min, 4°C. The supernatant was extracted as the nuclear fraction.

### Western blot analysis

Equal amounts of proteins were size-fractionated using SDS-polyacrylamide gel electrophoresis and electrotransferred onto polyvinylidene difluoride transfer membranes (Millipore). Blots were incubated for 1 hr at room temperature in 7% BSA for blocking, and proteins were detected with primary antibodies overnight, and then blotted with horseradish peroxidase conjugated secondary antibodies for 45 min. The immunoblots were visualized using ECL (Millipore). The mouse monoclonal antibody against cytochrome c was provided from ZYMED Laboratories, Inc. (South San Francisco, CA, USA). All other antibodies were purchased from Santa Cruz.

### Xenograft assays in nude mice

The animal experiments were approved by the Committee on the Ethics of Animal Experiments of the Xiamen University Medical College and carried out in the Animal Research Center, Department of Basic Medicine. HepG2 cells (2 × 10^6^) were implanted by subcutaneous injection into the right foreleg of the female Balb/c nude mice. The mice in the 3 treatment groups received intraperitoneal (i.p.) injections of 100 μL matrine at dose of 50, 75 or 100 mg/kg. Meanwhile, the mice in the negative and positive groups received i.p. injection of 100 μL of physiological saline and cyclophosphamide at 50 mg/kg, respectively. The mice were monitored every day, weighed before received i.p. injection of matrine, and killed 3 weeks after treatment, and then the tumors were used to do further experiments. Nine nude mice were used in each group of experiment.

### Immunohistochemical staining

Tumor tissue specimens were fixed in neutral formalin and embedded in paraffin after collection from the killed mice. Tissue Sections 5 μm thick were dewaxed and incubated with 0.01 M natrium citricum for antigen retrieval. The slides were rinsed in PBS and incubated overnight at 4°C with diluted anti-AIF antibodies. Following experiments were performed using the immunostaining kit according to the manufacturer’s instructions.

### Statistic analysis

All data are showed as the means ± S.D. for at least three separate determinations for each group. The differences between the groups were examined for statistical significance using the Student’s t-test with Prism 5 software.

## Abbreviations

AIF: Apoptosis inducing factor; MNNG: N-methyl-N’-nitro-N-nitrosoguanidine; PCD: Program cell death; ciPCD: Caspase-independent PCD; ΔΨm: Mitochondrial transmembrane potential; ROS: Reactive oxygen species; HCC: Hepatocellular carcinoma; HBV: Hepatitis B virus; HCV: Hepatitis C virus; z-VAD-fmk: Carbobenzoxy-valyl-alanyl-aspartyl-[O-methyl]-fluoromethylketone; DAPI: 4’,6-diamidino-2-phenylindole.

## Competing interest

The authors declare that they have no competing interests.

## Authors’ contributions

TH, YZ and GS designed the experiments and wrote the manuscript. HZ, MX, YG, ZD, WZ and HC performed the experiments, BZ analyzed the data. All authors read and approved the final manuscript.

## Supplementary Material

Additional file 1: Figure S1 Matrine induced caspase-dependent and -independent cell death in HepG2 cells. (A) Cells were treated with different concentrations of matrine (0, 0.25, 0.5, 1, 1.5, 2 mg/ml) for 24 hrs, or 1.0 mg/ml matrine for different time periods (0, 12, 24, 36, 48 hrs), and then the cell death was determined by annexin V/PI staining assay. (B) Cells were collected after treated with different concentration of matrine for 24 hrs and analyzed for ΔΨm by Rh123 retention. (C) Effect of pancaspase inhibitor, z-VAD-fmk, on matrine-induced cell death. HepG2 cells were pretreated with z-VAD-fmk (20 μM, 2 hrs) before matrine treatment. Cell death was analyzed by PI staining assay. Click here for file

Additional file 2: Figure S2 HepG2 cells were transfected with AIF siRNA (40 or 60 nM) or non-targeted siRNA for 24 hrs, and then treated with matrine at 1.5 mg/ml for 24 hrs. Cells were stained with propidium iodide (Annexin V/PI) to assess cell viability by flow cytometry. Click here for file

Additional file 3: Figure S3 Bid mediated the cell death induced by matrine, as an upstream regulator of AIF. HepG2 cells were transfected with Bid siRNA (40 or 60 nM) or non-targeted siRNA for 24 hrs (A) or pretreated Bid inhibitor BI-6C9 (10 μM) for 1 hr (B), then treated with matrine at 1.5 mg/ml for 24 hrs. Cells were stained with Annexin V/PI to assess cell viability by flow cytometry. Click here for file

Additional file 4: Figure S4 ROS production is required for AIF activation in HepG2 cells. (A, B) Cells were treated with matrine at different concentrations (0, 0.25, 0.5, 1, 1.5, 2 mg/ml) for 24 hrs or at 1.5 mg/ml for 24 hrs in the absence or presence of Tiron (5 μM), the ROS scavenger. ROS levels were then detected by flow cytometry. (C) Tiron significantly attenuated matrine-induced cell death as determined by Annexin V/PI staining and flow cytometry. Click here for file
