# Supplementary figures and images for: Matrine induces caspase-independent program cell death in hepatocellular carcinoma through bid-mediated nuclear translocation of apoptosis inducing factor
Source: Mol Cancer. 2014 Mar 16;13:59. doi: 10.1186/1476-4598-13-59 (PMC4007561; doi:10.1186/1476-4598-13-59)

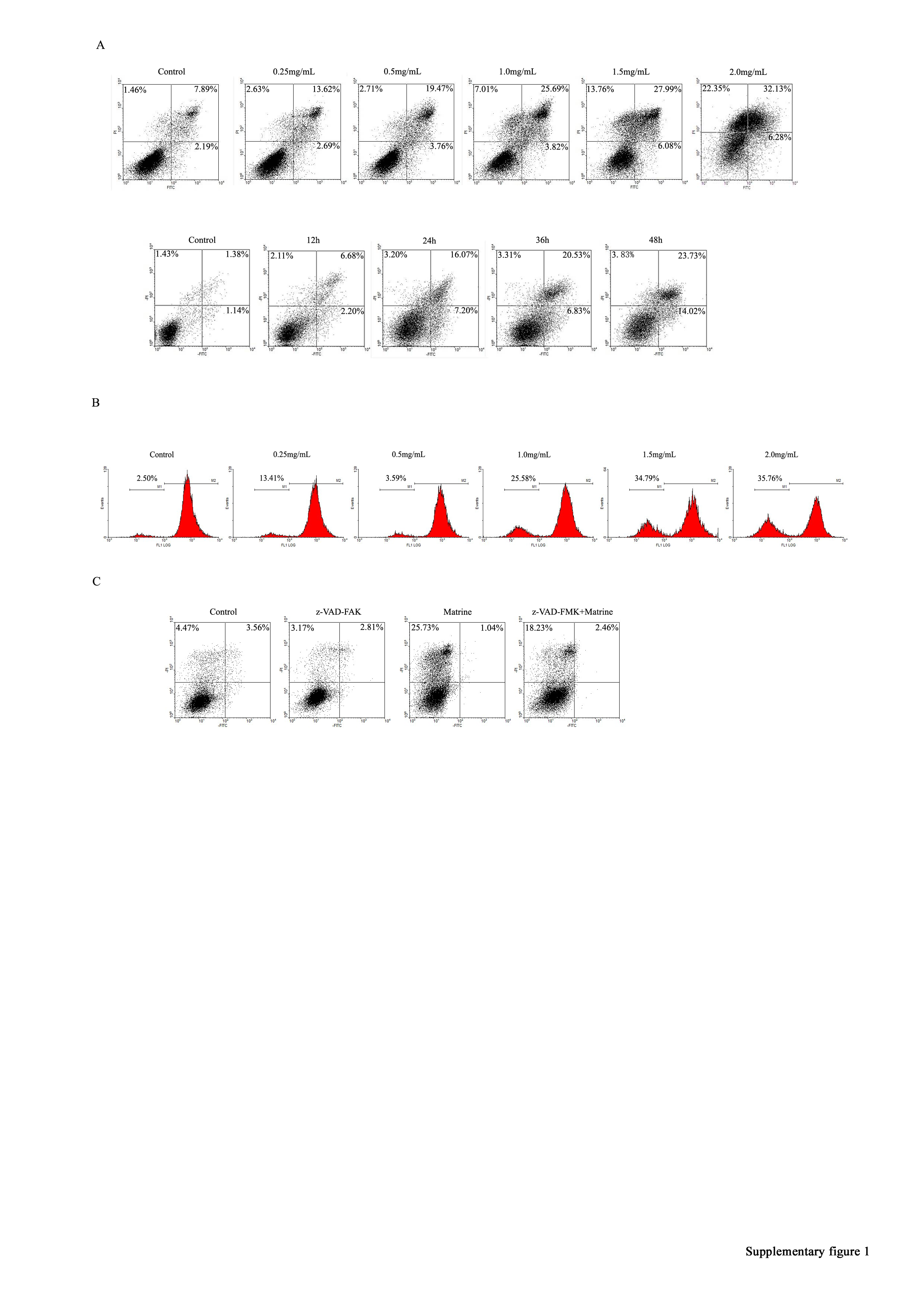

Supplement: Additional file 1: Figure S1 — Matrine induced caspase-dependent and -independent cell death in HepG2 cells. (A) Cells were treated with different concentrations of matrine (0, 0.25, 0.5, 1, 1.5, 2 mg/ml) for 24 hrs, or 1.0 mg/ml matrine for different time periods (0, 12, 24, 36, 48 hrs), and then the cell death was determined by annexin V/PI staining assay. (B) Cells were collected after treated with different concentration of matrine for 24 hrs and analyzed for ΔΨm by Rh123 retention. (C) Effect of pancaspase inhibitor, z-VAD-fmk, on matrine-induced cell death. HepG2 cells were pretreated with z-VAD-fmk (20 μM, 2 hrs) before matrine treatment. Cell death was analyzed by PI staining assay. [file 1476-4598-13-59-S1.tiff]

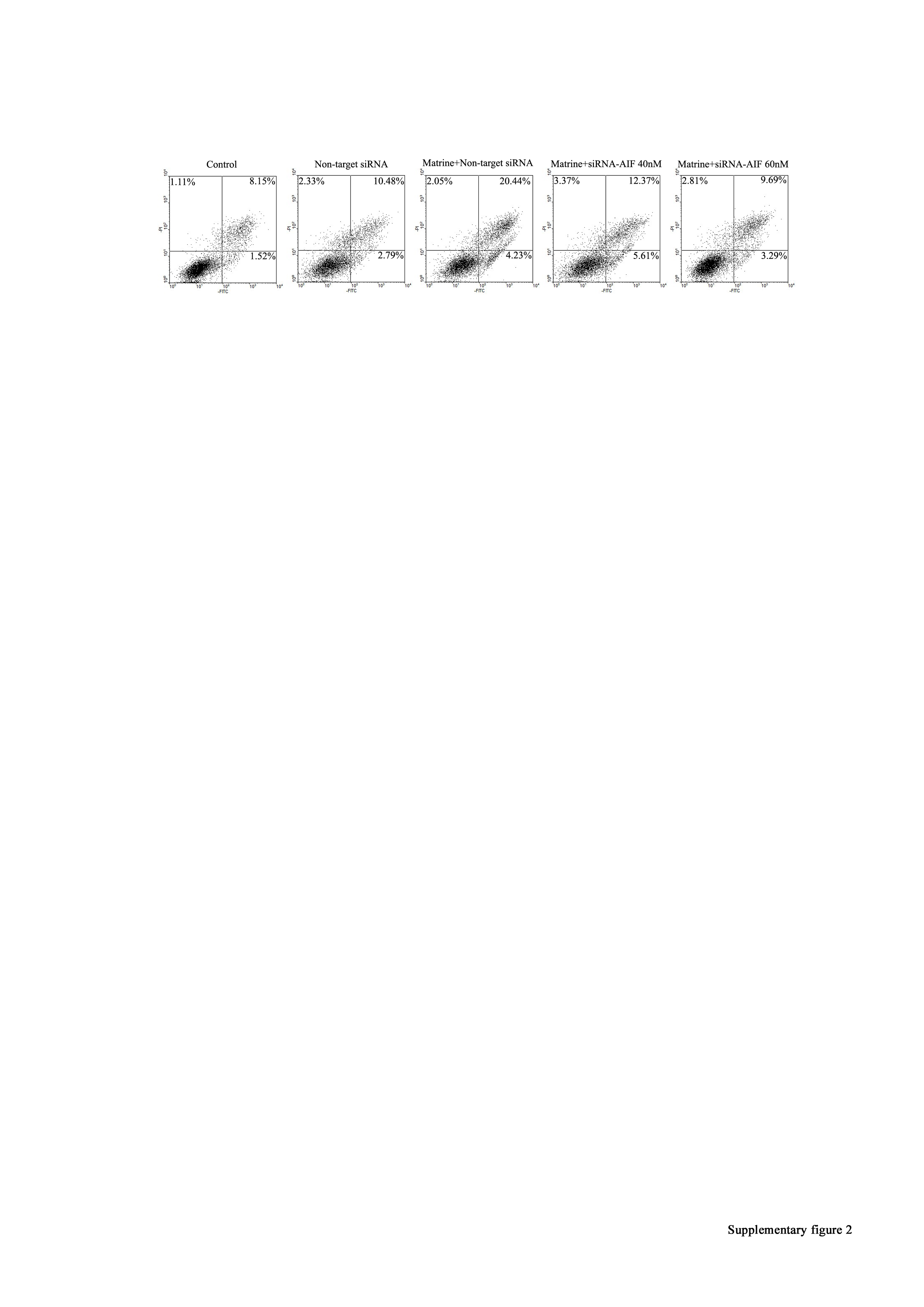

Supplement: Additional file 2: Figure S2 — HepG2 cells were transfected with AIF siRNA (40 or 60 nM) or non-targeted siRNA for 24 hrs, and then treated with matrine at 1.5 mg/ml for 24 hrs. Cells were stained with propidium iodide (Annexin V/PI) to assess cell viability by flow cytometry. [file 1476-4598-13-59-S2.tiff]

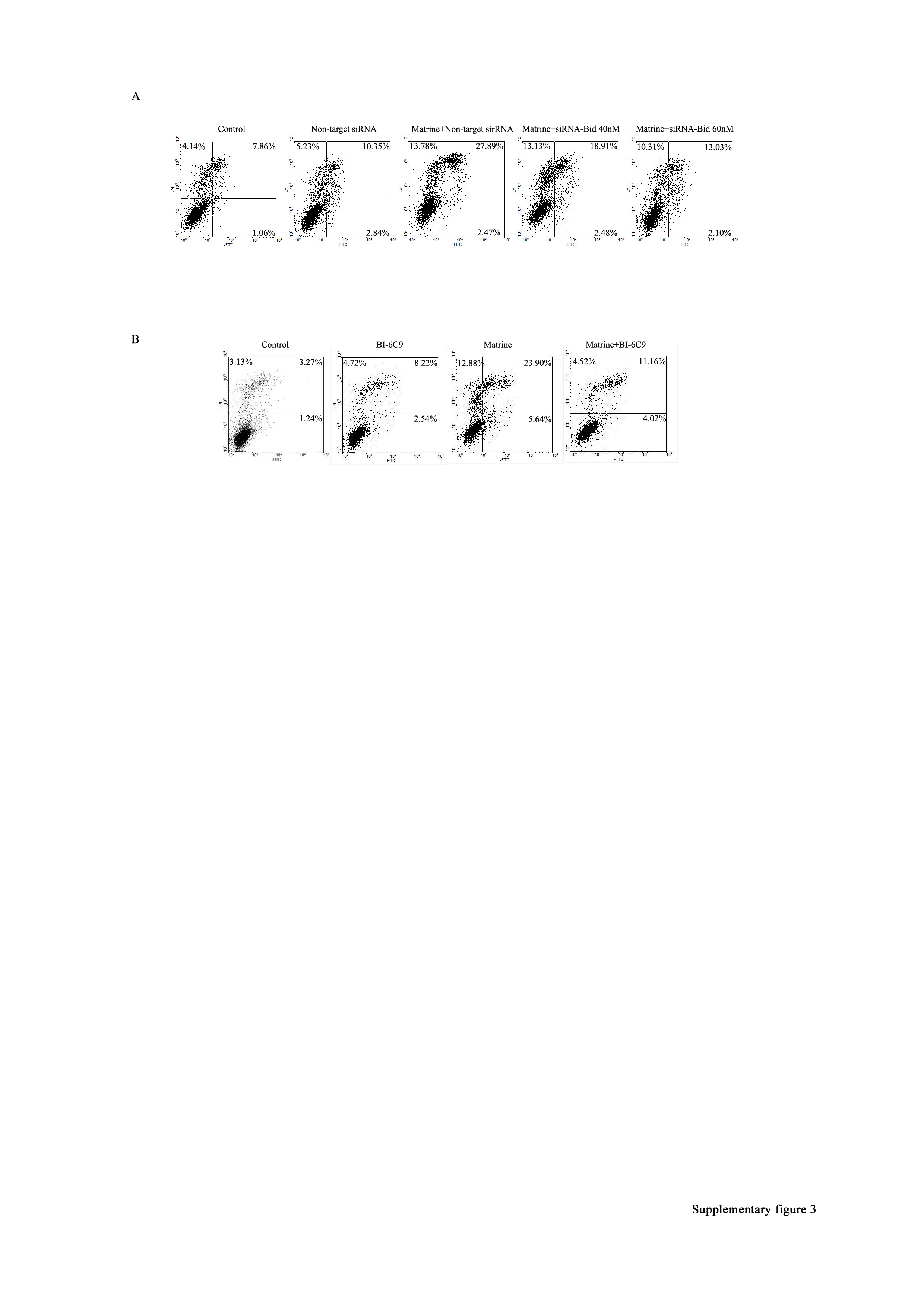

Supplement: Additional file 3: Figure S3 — Bid mediated the cell death induced by matrine, as an upstream regulator of AIF. HepG2 cells were transfected with Bid siRNA (40 or 60 nM) or non-targeted siRNA for 24 hrs (A) or pretreated Bid inhibitor BI-6C9 (10 μM) for 1 hr (B), then treated with matrine at 1.5 mg/ml for 24 hrs. Cells were stained with Annexin V/PI to assess cell viability by flow cytometry. [file 1476-4598-13-59-S3.tiff]

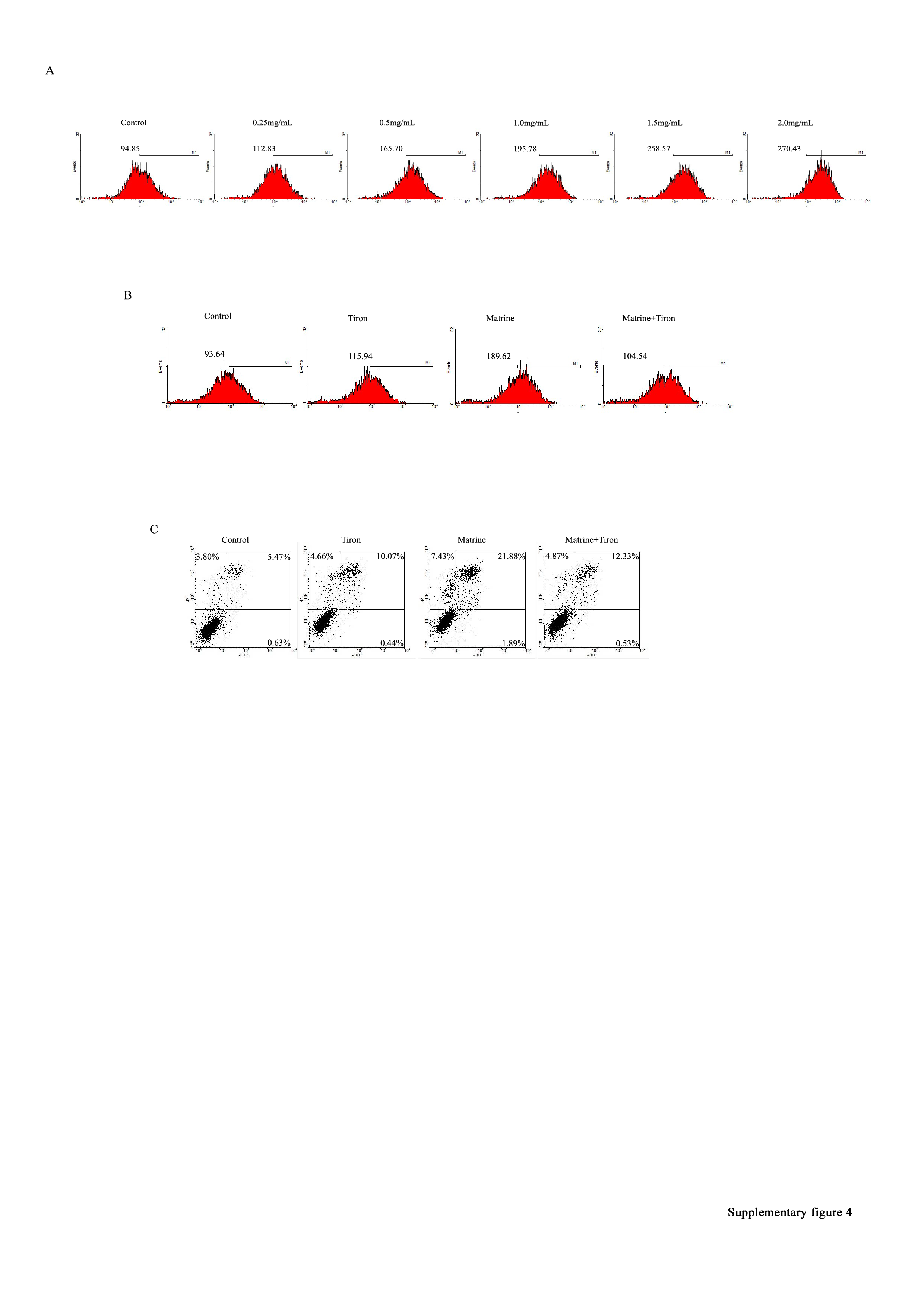

Supplement: Additional file 4: Figure S4 — ROS production is required for AIF activation in HepG2 cells. (A, B) Cells were treated with matrine at different concentrations (0, 0.25, 0.5, 1, 1.5, 2 mg/ml) for 24 hrs or at 1.5 mg/ml for 24 hrs in the absence or presence of Tiron (5 μM), the ROS scavenger. ROS levels were then detected by flow cytometry. (C) Tiron significantly attenuated matrine-induced cell death as determined by Annexin V/PI staining and flow cytometry. [file 1476-4598-13-59-S4.tiff]
